# Human Challenge Studies to Accelerate Coronavirus Vaccine Licensure

**DOI:** 10.1093/infdis/jiaa152

**Published:** 2020-03-31

**Authors:** Nir Eyal, Marc Lipsitch, Peter G Smith

**Affiliations:** 1 Center for Population-Level Bioethics, Rutgers University, New Brunswick, New Jersey, USA; 2 Department of Philosophy, Rutgers University, New Brunswick, New Jersey, USA; 3 Department of Health Behavior, Society and Policy, Rutgers School of Public Health, Piscataway, New Jersey, USA; 4 Center for Communicable Disease Dynamics, Department of Epidemiology, Harvard T. H. Chan School of Public Health, Boston, Massachusetts, USA; 5 Department of Immunology and Infectious Diseases, Harvard T. H. Chan School of Public Health, Boston, Massachusetts, USA; 6 MRC Tropical Epidemiology Group, London School of Hygiene & Tropical Medicine, London, UK

**Keywords:** coronavirus, vaccines, human challenge studies, randomized controlled trials, risk-taking, ethics

## Abstract

Controlled human challenge trials of SARS-CoV-2 vaccine candidates could accelerate the testing and potential rollout of efficacious vaccines. By replacing conventional phase 3 testing of vaccine candidates, such trials may subtract many months from the licensure process, making efficacious vaccines available more quickly. Obviously, challenging volunteers with this live virus risks inducing severe disease and possibly even death. However, we argue that such studies, by accelerating vaccine evaluation, could reduce the global burden of coronavirus-related mortality and morbidity. Volunteers in such studies could autonomously authorize the risks to themselves, and their *net* risk could be acceptable if participants comprise healthy young adults, who are at relatively low risk of serious disease following natural infection, if they have a high baseline risk of natural infection, and if during the trial they receive frequent monitoring and, following any infection, the best available care.

## Teaser

Controlled human challenge trials of SARS-CoV-2 vaccine candidates could accelerate the testing and potential rollout of efficacious vaccines, reducing the global burden of coronavirus-related mortality and morbidity. Volunteers’ net risk in such studies could be acceptable under specific conditions.

**Figure 1.**
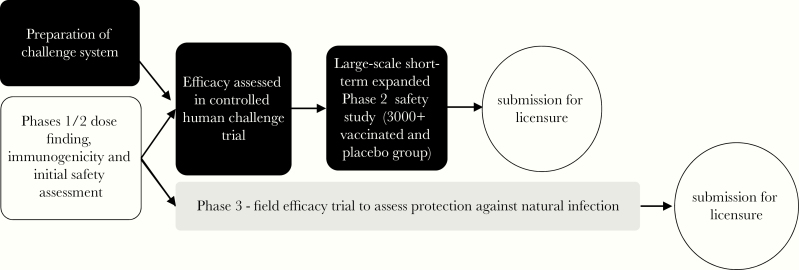
The process to vaccine licensure through a controlled human challenge trial and large study to assess short-term safety (black) compared to the conventional phase 3 trial route to licensure (grey). Submission for licensure could occur substantially earlier with a controlled human challenge trial.

Alleviation of the enormous burden of mortality and morbidity associated with the COVID-19 pandemic will probably depend on the development of effective vaccines that could be rolled out widely. Many candidate vaccines are in development [1], but recent estimates cite at least 1–1.5 years to vaccine rollout [2]. A significant proportion of that time is due to the requirement to assess efficacy and safety in placebo-controlled phase 3 trials, which typically involve several thousand participants followed for long enough in the field to assess differences in disease incidence between vaccine and control groups, with many participants taking precautions to avoid exposure. We suggest that, in the circumstances of a devastating global pandemic, controlled human challenge studies (following the normal initial safety, vaccine dose finding, and immunogenicity studies—phases 1/2 in Figure 1) may be an acceptable way to bypass phase 3 testing, and speed the licensure of efficacious vaccines.

## THE PROPOSED STUDY DESIGN

Volunteers for human challenge studies would be drawn from previously uninfected individuals at relatively low risk of complications or mortality from SARS-CoV-2 infection (eg, young adults, without chronic health conditions, and not otherwise sick) [3–7] and who are at substantial risk of natural exposure to SARS-CoV-2 (eg, resident in areas with high transmission rates). Such a target group might comprise uninfected persons aged 20–45 years, an age range in which the risk of death or serious complications following infection is substantially lower than in older age groups [4, 5].

The controlled challenge model would need to be standardized before using it to test vaccines. Volunteers, previously uninfected, would be required for an initial dose-escalation study of the viral challenge to select a dose of virus exposure such that most placebo recipients become infected (for statistical reasons), and have a clinical response that is not more severe than the one associated with natural infection (for ethical reasons). The latter would require comparison with a cohort of individuals of similar age who had been infected naturally. For this standardization, volunteers may spend 2 weeks in a clinical isolation facility prior to the challenge, with viral and serologic testing, to exclude those with previous or recent infection (or a shorter duration if suitable serological tests for recent infections are developed). Overall, these preparatory studies (upper-left black square in Figure 1) may take several weeks and could start before vaccine candidates are available for evaluation.

Multiple measures would be put in place to ensure that, prior to consenting, potential participants fully comprehend the unusual risks involved in the study.

After the controlled human challenge model had been set up, vaccines could be evaluated. Volunteers who had not been previously infected would be randomized to receive either the candidate vaccine(s) under investigation or placebo. After an interval to permit a full immune response to the vaccine, a controlled exposure to SARS-CoV-2 would be administered. Appropriate vaccine schedules (eg, dose, number of doses) will have been determined, to the extent possible, in the conventional phase 1/2 immunogenicity and safety studies that preceded the challenge study.

Following the challenge, the participants would be carefully followed, to monitor whether those vaccinated had a different response to viral challenge. Because the challenge studies would be relatively small and some volunteers might show few clinical symptoms, there would need to be careful consideration of the choice of the primary endpoint, through discussions with regulatory authorities. Possibilities would include viral load, measured at least daily (eg, in throat swabs), and then cumulated over the course of the infection (as has been done in influenza challenge studies) [8], and time to first clinical symptoms. For some vaccines, the endpoint might be the proportion infected. Throughout the study, intense immunological monitoring would seek any correlates of vaccine effect. The required size of such studies would depend upon the endpoints chosen, but they might require of the order of 100 volunteers.

Any volunteers in whom infection was confirmed would receive excellent care for COVID-19, including priority for any scarce life-saving resources, in state-of-the-art facilities. Throughout the trial and until infectiousness was ruled out, all participants would remain isolated in a secure and comfortable setting (eg, in settings converted from those used for influenza challenge studies).

If this human challenge study showed a vaccine candidate to be efficacious, an expanded placebo-controlled study would be conducted in the field, involving at least 3000 vaccinated persons, primarily for short-term safety assessment, but also to gather further evidence on immunogenicity (Figure 1, right-most black box). Participants would be carefully monitored for adverse effects following vaccination, to gather safety data sufficient for submission for licensure. This study (not involving a challenge) should be conducted on the eventual primary initial target group for an effective vaccine—including the elderly and those with concomitant illnesses that increase the risk of serious disease following infection. With prior planning, this large-scale assessment of safety could be completed in several months, as initially only short-term adverse effects would be assessed.

Together, the information from the challenge study and the short-term follow-up of those in the expanded (phase 2) field study may produce evidence sufficient to justify accelerated licensure.

Participants of the expanded field study could continue to be followed longer term in parallel with the submission for licensure, so that suitable actions could be taken if any long-term adverse effects, including disease enhancement, were identified. As with standard vaccine licensure, additional, postapproval studies would be required to assess safety and effectiveness in routine use. Any necessary studies of dosage and safety in special groups (eg, children, pregnant women, and immunocompromised persons) could be conducted, before extending vaccination to these groups, as judged appropriate.

It is possible that the protection that was apparent in a challenge study will not be replicated when the vaccine is used to protect against natural infection. This would have to be carefully monitored in the early stages of vaccine rollout, for example through case-control studies. In such an event, appropriate modification will be made to the vaccination program (including potentially stopping vaccination).

A particular concern with respect to some vaccine constructs against coronavirus is that they may induce more severe disease following infection, as has been reported in animal models of both SARS and MERS vaccine candidates [9]. If any vaccine candidate shows evidence of such effects in animal models, it is likely to be ruled out for human testing. However, for those candidates that are taken forward for human testing, the possibility of enhancement should be borne in mind and the challenge studies should be designed in such a way that small groups of volunteers are challenged sequentially. In this way, studies could be stopped at an early stage, upon first strong indication of vaccine-induced enhanced disease. If the vaccine candidate did enhance disease, the controlled human challenge model would provide much more rapid evidence to support stopping the testing of a harmful vaccine candidate, with far fewer vaccinated persons, than a traditional phase 3 efficacy study.

## ACCELERATION OF LICENSURE AND SUBSTANTIAL SOCIAL VALUE

The proposed trial method would potentially cut the wait time for the rollout of an efficacious vaccine. Challenge studies (which always directly expose all participants to a pathogen to assess efficacy) generally require fewer participants, followed over a shorter period than do standard efficacy studies (in which many participants are never exposed). Rollout of an efficacious vaccine to age groups not included in the challenge studies may depend on immunological bridging, but this would be a component of the expanded safety studies discussed above. It is possible that this process could take several months shorter than reliance on standard phase 3 testing to assess efficacy. While rollout to other populations might require initial bridging studies, these could be conducted relatively quickly.

It seems clear that, in the absence of an efficacious vaccine, the global death toll from COVID-19 will be enormous. A recent modelling study suggests that, even with mitigation strategies focusing on shielding the elderly and slowing but not interrupting transmission, there may be 20 million this year [10]. If the use of human challenge helped to make the vaccine available before the epidemic has completely passed, the savings in human lives could be in the thousands or conceivably millions. Intense social distancing and related control measures, held in place for many months between now and the availability of vaccine, will themselves take a toll on economies, societies, and population health. Advancing the registration and rollout of an efficacious vaccine, even by a few months, could save many thousands of lives, and commands enormous societal value.

## AUTONOMOUS AUTHORIZATION

Deliberate exposure of study participants to SARS-CoV-2 gives rise to understandable ethical worries. It may seem impermissible to ask people to take on risk of severe illness or death, even for an important collective gain. But we actually ask people to take such risks for others’ direct gain every time we ask volunteer firefighters to rush into burning buildings, relatives to donate a live organ to loved ones, healthy volunteers to participate in drug and vaccine toxicity trials with no prospect of improving their health (and some risk of undermining it) [11], relatively healthy volunteers to participate in studies involving long antiretroviral drug interruptions that risk their health with negligible prospect of improving it [12], and other challenge studies in which healthy volunteers expose themselves to pathogens [13]. This spring, we are clearly within our right when we invite citizens to volunteer for Emergency Medical Services (EMS) to fight a pandemic that augments both the personal risks for EMS workers and the social value of their work, and initial trials for the Moderna SARS-CoV-2 vaccine are being accelerated by skipping prior animal testing and the margin of safety that it would have added [14].

One major reason why it is permissible to risk medical harm to volunteers in medical studies, even when their personal health care does not require that risk, is that these volunteers will have autonomously consented to take on these risks. Adult persons can legitimize many interventions in their bodies and health that are normally prohibited, simply by saying “Yes,” with full understanding and voluntariness. In the present case, the study would involve multiple tests of comprehension of all risks (and the risk factors for serious outcomes among individuals in otherwise low-risk age groups may be somewhat clearer by the time recruitment takes place), so that the decision is deeply informed and voluntary. The exclusive recruitment of participants aged 20–45 years, although children are less likely to have severe symptoms upon COVID-19 infection [6, 7], seeks to safeguard the quality of participants’ consent. The wide news coverage and widespread fear of Covid-19 should keep it clear that exposure to this virus is no small matter. While in other studies mentioned above, nonconsenting sex partners and fetuses of study participants may get infected [15, 16], the proposed controlled challenge study would avoid risk to nonparticipants by isolating participants whilst infectious.

## ADDED RISK REMAINS ACCEPTABLE

But a remaining key question, for deeming human challenge studies ethical, pertains to risk. Are the risks to participants, even when they are justified by the social importance of the trial and backed by participants’ willful permission, also being kept to the necessary minimum? And do the risks fall below a postulated cap on the acceptable risk of medical trials, even ones of the highest social value and with participants’ consent [15]?

The proposed challenge studies seek to contain the risk to participants in 6 different ways. First, the study will recruit only healthy patients from age groups in which the risk of severe disease and death following SARS-CoV-2 infection is low. Second, there is the possibility that the vaccine candidate will protect at least some of those who are vaccinated. Third, in the absence of an effective vaccine, a high proportion of the general population is likely to be naturally infected with SARS-CoV-2 at some point [17], including those who might participate in a challenge study; by volunteering to be artificially infected they may be just hastening an event that is likely to occur in later months anyhow. Fourth, only people with an especially high baseline risk of getting exposed during or soon after the trial period should be recruited (eg, people residing in areas with high transmission rates). Fifth, participants would be monitored carefully and frequently following the challenge and afforded the best available care if needed (eg, guaranteed access to state-of-the-art facilities of the health system, notwithstanding the possibility of severe shortages of medical care during the evolving pandemic). Sixth, by the time vaccine candidates are being tested, some therapeutics may be approved, which may reduce participants’ risk of morbidity and mortality further. For these 6 reasons, mortality and morbidity from participation notwithstanding, *net* mortality and morbidity from participation should remain low or negative.

## CONCLUSION

A novel strain of coronavirus forces us to consider unconventional approaches. We believe that controlled SARS-CoV-2 vaccine challenge studies may accelerate the time it takes to evaluate and license vaccines and hence could make vaccines available sooner for widespread rollout. Such an approach is not without risks, but every week that vaccine rollout is delayed will be accompanied by many thousands of deaths globally. Importantly, challenge studies are conducted against the background of competent volunteers’ informed consent, minimization of study risks, and high baseline risks of infection for participants. They do not violate participants’ individual rights on the altar of emergency response, but heed both individual rights and the global public health emergency.

As far as we are aware, the current plan for evaluating the efficacy of COVID-19 vaccines is through slower phase 3 trials. It will take some time to develop and operationalize challenge studies and we are not suggesting that ongoing development of any vaccines which become ready for phase 3 trials must be paused during this period. However, we believe that challenge studies could be set up before the time that most vaccines would be ready for efficacy testing.

In addition to their use for assessing the efficacy of vaccines, human challenge studies may also help evaluate drugs that might be given either as preexposure prophylaxis to prevent infection in individuals at high risk of infection, or as postexposure prophylaxis, given shortly after a potential exposure, either to abort infection or to prevent the occurrence of disease. Challenge studies may also be a way of advancing understanding of the pathogenesis of the progression from infection to disease.

To further assess the potential of human challenge studies to speed vaccine development, we suggest that an expert group might be convened, including those with experience of human challenge studies of other pathogens, regulators, vaccine trialists, ethicists, potential participants, and relevant funding agencies, to plan if and how such studies might be taken forward ethically and expeditiously.

## Notes

***Disclaimer.*** The views expressed in this publication are those of the authors and not necessarily those of the Department of Health and Social Care or the US National Institutes of Health.

***Financial support***. This work was supported by the National Institute of Allergy and Infectious Diseases (grant number AI114617-01A1 to N. E.); National Institute of General Medical Sciences (grant number U54GM088558 to M. L.); M. L.’s work is supported by the Morris-Singer Foundation, and the Department of Health and Social Care with UK Aid funding managed by the National Institute for Health Research; P. G. S.’s work is partly funded by the UK Medical Research Council (MRC) and the UK Department for International Development (DFID) under the MRC/DFID Concordat agreement; and the European Union (EDCTP2 program).

***Potential conflicts of interest.*** M. L. has received honoraria from Merck. N. E. and P. S. report no potential conflicts of interest. All authors have submitted the ICMJE Form for Disclosure of Potential Conflicts of Interest. Conflicts that the editors consider relevant to the content of the manuscript have been disclosed.
